# A simple and low-cost combustion method to prepare monoclinic VO_2_ with superior thermochromic properties

**DOI:** 10.1038/srep39154

**Published:** 2016-12-15

**Authors:** Ziyi Cao, Xiudi Xiao, Xuanming Lu, Yongjun Zhan, Haoliang Cheng, Gang Xu

**Affiliations:** 1 Key Laboratory of Renewable Energy, Chinese Academy of Sciences; Guangdong Key Laboratory of New and Renewable Energy Research and Development, Guangzhou Institute of Energy Conversion, Chinese Academy of Sciences, Guangzhou 510640, P. R. China; 2 Nano Science and Technology Institute, University of Science and Technology of China, Suzhou 215123, P. R. China; 3 University of Chinese Academy of Sciences, Beijing 100049, P. R. China

## Abstract

In this approach, the VO_2_ nanoparticles have been successfully fabricated via combusting the low-cost precursor solution consisted of NH_4_VO_3_, C_2_H_6_O_2_ and C_2_H_5_OH. By the XRD, TEM and XPS analysis, it can be found that the synthetic monoclinic VO_2_ is single crystal and no impurity is defined. After dispersing the VO_2_ nanoparticles into the polymer, the solar modulation of VO_2_-based composite film is up to 12.5% with luminous transmission and haze around 62.2% and 0.5%, respectively. In other words, the composite films show high performance of thermochromic properties. This could open an efficient way to fabricate low-cost and large-scale VO_2_ (M) nanoparticles and thermochromic films.

Monoclinic vanadium dioxide VO_2_ (M) which was reported firstly by Morin in 1959^1^, has attracted significant attention due to its superior physico-chemical property. The reversible and abrupt semiconductor-metal transition could be associated with the change of crystallographic structure from the monoclinic (M-phase, P21/c, semiconductor) to the tetragonal (R-phase, P4_2_/mnm, mental) phase at 68 °C. And dramatic changes in IR region transmission and electrical resistance also occurred simultaneously. By these advantages, VO_2_ (M) is believed to be a potential functional material for various industrial and technological applications such as electrical devices^2^,^3^ and energy-efficient smart windows^4^,^5^.

In the past several decades, different methods including sputting deposition^6^, chemical vapour deposition(CVD)^7^, sol-gol^8^,^9^,^10^, pyrolysis^11^,^12^ and hydro/solvothermal^13^,^14^,^15^,^16^,^17^ have been developed for preparing VO_2_ (M). However, these methods suffer from some limitations such as harsh gas atmosphere with exactly controlled flow, time consuming or post-treatment with high-temperature. In order to enable a practical process for the application of VO_2_ (M) in industrial fields, developing simple and efficient methods are highly desired.

Recently, there are several groups which report some accessable methods to prepare VO_2_ (M). Y. Xie^18^ *et al*. have studied in detail that the several different precursors to prepare VO_2_ by the combustion method. VO(acac)_2_ and Bis (α-furylacrylic acid) oxovanadium (IV) complex is considered as the V source, which could fabricate VO_2_ nanoparticles. However, the Bis (α-furylacrylic acid) oxovanadium (IV) complex needs to be prepared by the time-consuming process, of which the yield rate is also low^19^. And the price of VO(acac)_2_ is more expensive. In 2013, G.T. Chandrappa^20^ *et al*. developed a solution consisted of NH_4_VO_3_ and DL-malic acid to prepare monoclinic VO_2_ by the combustion. The VO_2_ can be obtained in 5 min, which is quite fast to collect VO_2_ nanopowder. Unfortunately, the method needs 470 ± 10 °C in muffle furnace to guarantee enough energy to form VO_2_. In addition, at the smouldering stage, the air partial pressure is not easy to be controlled exactly, which is not helpful for the formation of the pure VO_2_ (M). J. Zou and co-workers^21^ have reported the thermolysis of VEG (vanadyl ethylene glycolate) for synthesizing VO_2_ (M) in air. Nevertheless, the multi-step operation and time-consuming process would limit its practical application. By contrast, further works should be made for providing a facile route to prepare VO_2_ (M), especially the production of VO_2_ (M) nanoparticles in a large scale and promotion of thermochromic smart window in a low cost.

As part of an ongoing interest on preparation of VO_2_ (M), we herein propose a simple and low-cost method for preparing VO_2_ (M) nanoparticles with high purity and thermochromic performance. This process only involves the combustion of a mixture of EG (ethylene glycol), NH_4_VO_3_ and EtOH (ethyl alcohol) in several minutes. Compared with the previous methods, the VO_2_ (M) could be obtained with high cost performance. The thermochromic performance of VO_2_-based composite film shows 12.5% for the solar transmittance modulation with luminous transmission and haze around 62.2% and 0.5%, respectively. These privileges make it be possible for the production of VO_2_ (M) and thermochromic films in a large-scale with low cost.

## Results and Discussion

### Fabrication mechanism of the VO_2_ (M)

**Figure 1:**
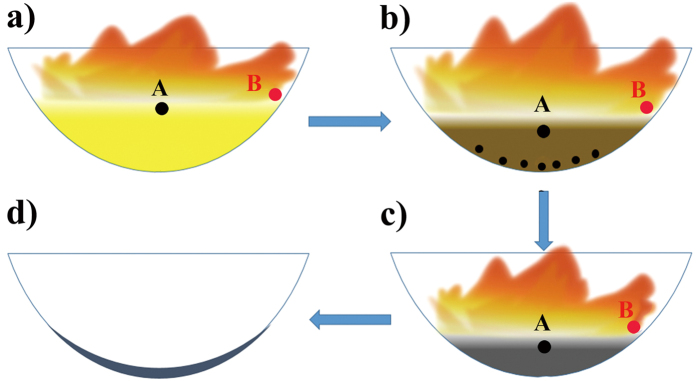
Schematic illustration of the combustion in evaporating dish: (**a**) 0 min; (**b**) 6 min; (**c**) 9 min; (**d**) 18 min.

**Figure 2:**
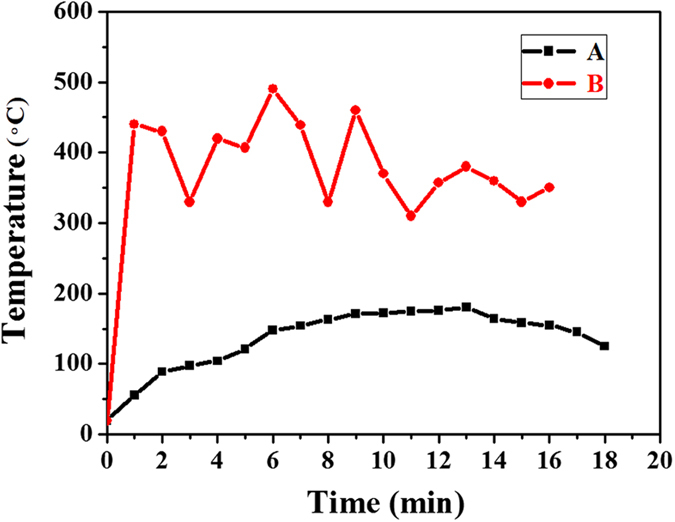
The curve A, B corresponding to the evolution of the temperature at position A, B, respectively.

In this approach, VO_2_ (M) can be formed *via* a solution combustion process as illustrated in Fig. 1, followed by all-around characterization. In order to explore reaction mechanism, the temperature evolution of position A, B in Fig. 1 directly measured by the thermocouple are shown in Fig. 2. The position A is located at the interface of the flame and solution. The position B is located at the interface of the flame, solution and inner wall of the container. The temperature measurement at position A is to prove the formation of VEG. The temperature at position B is variable on account of dynamic change of flame, whereas the change trend of temperature could be used to analyze the temperature scale of position B in a full reaction process.

From the temperature evolution in Fig. 2, it can be found that at 6 minutes, the temperature of position A is up to 120 °C and kept at the above 120 °C over a period of time, which ensures the formation of VEG by reaction between NH_4_VO_3_ and C_2_H_6_O_2_^21^. And at the same time, the colour of the solution changes from bright yellow to brown and a little precipitates appear on the inner wall of container as shown in Fig. 1b. The temperature at position A is increased to 171 °C at 9 minutes, and a black reaction solution is observed (in Fig. 1c). In order to clarify this change, the reaction was interrupted at the time and the resulting precipitates were collected.

**Figure 3:**
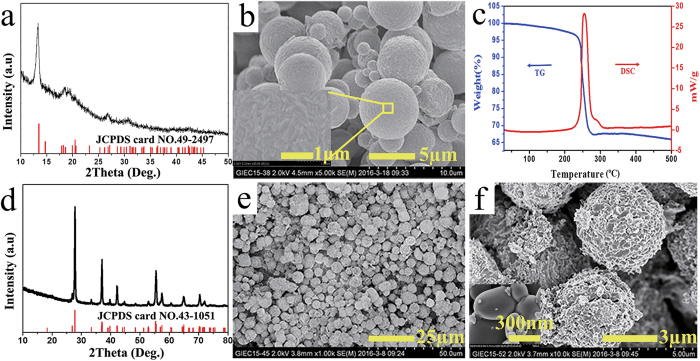
(**a**) XRD pattern and (**b**) SEM image of the precipitate obtained at 9 min; (**c**) TG and DSC curves of the precursor VEG; (**d**) XRD pattern and (**e–f**) SEM images of the black-blue powder.

The XRD pattern of the precipitate is shown in Fig. 3a. It can be found that the XRD pattern of the precipitate matches well with that of the standard JCPDS card (No. 49-2497) corresponding to VEG. No peaks of other phases and impurities are observed, which indicates that pure VEG could be produced in the reaction as the following equation Eq. (1).


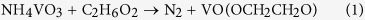


The morphology and microstructure determined by SEM are shown in Fig. 3b. It can be found that the VEG product is composed of dispersed microspheres with different sizes, which is similar with the previous results^22^. The surface structure of spherical VEG presents the aggregation of chain-like VEG as shown in the magnified SEM.

In order to explore the conversion process of the VEG, the thermal behavior of VEG in air is investigated subsequently. The TG and DSC curves of the VEG are shown in Fig. 3c. From the DSC curve, it can be found that there is an obvious exothermic peak around 253 °C, indicating that the decomposed temperature of the VEG is around 253 °C. The corresponding sharp mass loss can be found in TG curve. The total mass loss between 180 and 300 °C is 32.3%, which is approximate to the theoretical value of 34.6% calculated from the equation below^23^.


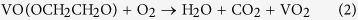


Hence, according to the TG and DSC curves, the heat treatment of the VEG at 300 °C can be sufficient for the complete decomposition. With the decreasing of solvent in the combustion process, more and more precipitates appear on the inner wall of the dish. The temperature of the position B is always above 300 °C in Fig. 2b. Therefore, the formation of VO_2_ proceeds at position B, resulting from the adequate energy and oxygen accessed. After reaction completion, the precipitates are fully converted into the black-blue powder.

The XRD pattern and SEM images of the black-blue powder are shown in Fig. 3d–f. It can be found that all diffraction peaks in XRD pattern are well in agreement with that of the standard JCPDS card (No. 43-1051) corresponding to VO_2_ (M). It means that after reaction the black-blue powder is VO_2_ (M). According to SEM image (Fig. 3e), the black-blue powder generated is the well-defined hierarchical spherical shape with diameters typically ranging from 3 to 8 μm. However, it can be observed that the microstructure consists of large quantities of nanoparticles from the magnified SEM image (Fig. 3f). The size of the nanoparticles is estimated to be 100–300 nm.

**Figure 4:**
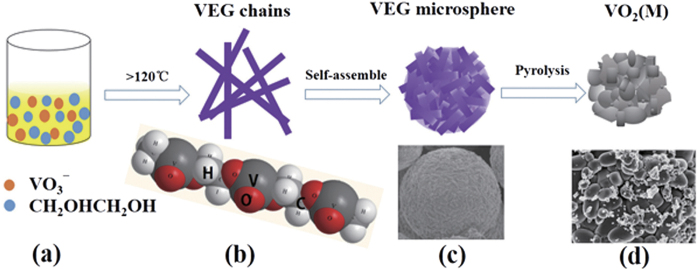
Schematic illustration of the formation mechanism of VO_2_ (M) nanoparticles.

As is well known, the molecular structure of VEG is a long chain^24^. Based on the reaction process and result obtained, it can be found that during the combustion, the chain-like VEG is formed and rapidly aggregate into spherical microparticles to reduce the total energy of the system as schematically illustrated in Fig. 4a–c. As the reaction proceeds, the formed VEG aggregates deposit on the inner wall. In addition, the temperature of position B is always above 300 °C, far higher than the decomposition temperature of VEG with 253 °C. The interface between flame and solution gradually drops with the solvent consumption. The deposited VEG on the inner wall can be decomposed directly at position B. Therefore, after combustion completion, the VEG precursor is decomposed *in-situ* and converted into VO_2_, which has been proved in our previous work as shown in the above equation (2)^23^. At the same time, the aggregated VO_2_ nanoparticles continue to grow to form big particles (in Fig. 4d) as a result of the evolution of CO_2_ gas, H_2_O vapor and structural rearrangement of VO_2_ units during the decomposition process, of which the result is similar with that of the literature^21^. Hence, the fabrication mechanism of VO_2_ (M) nanoparticles could be proposed as “precursor formation – self-assemble – pyrolysis” process (in Fig. 4).

**Figure 5:**
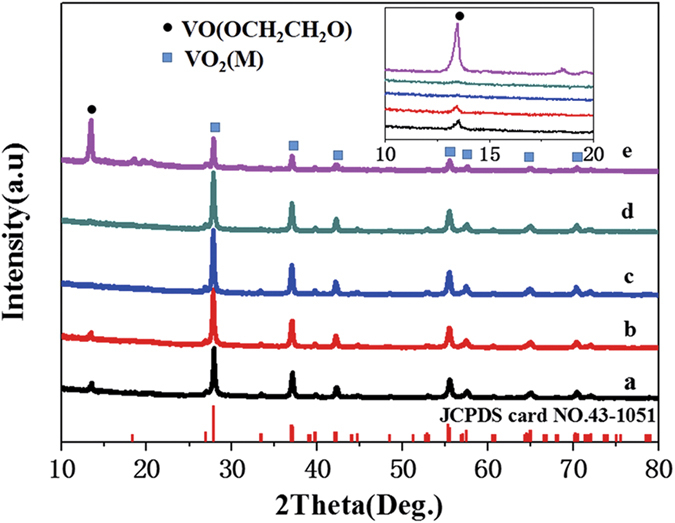
The XRD patterns of prepared sample in container with different curvature: (**a**) 0.23; (**b**) 0.20; (**c**) 0.17; (**d**) 0.13.

Taking into account the reaction performed in air, the curvature of the container could influence the reaction process. Thus, the containers with different curvature are investigated. The curvature radius and corresponding curvature of these containers can be seen from Table 1. The volume of reaction solution is kept at 100 mL. The XRD patterns of the products obtained from different containers are shown in Fig. 5. It can be found that with the change of curvature from 0.23 to 0 cm^−1^, the diffraction peak of VEG at 2θ = 13.6° shows the corresponding intensity and then disappears at curvature of 0.17 cm^−1^, and finally appears again (shown in the inset of Fig. 5). However, when the curvature is 0.17 cm^−1^, the diffraction peak for VO_2_ (M) at 2θ = 27.8° shows the strongest intensity and all diffraction peaks are ascribed to that of JCPDS 43–1051, with the lattice parameters a = 5.7529 Å, b = 4.5263 Å, c = 5.3825 Å, and β = 122.6°. No peaks of other phases or impurities are observed, indicating that the high purity of the VO_2_ (M) is obtained.

**Figure 6:**
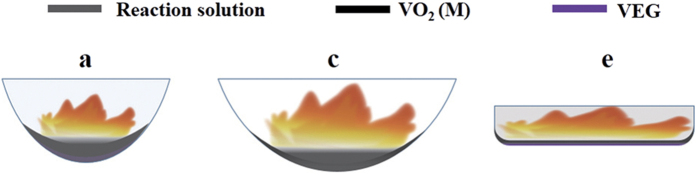
The combustion in the container (**a**), (**c**) and (**e**), respectively.

The possible reasons for the above results are hinted in Fig. 6. Due to the difference of curvature, the VEG precipitate layer in container (a) is thicker than (c). The VEG at the bottom could not be pyrolyzed sufficiently in the lack of oxygen and heat energy even though the reaction is exothermic. When the flame is quenched, therefore, little VEG is still existent at the bottom of the container (a) as shown in Fig. 6. Whereas the VEG precipitate layer in the container (e) is thinner than container (c), and the VEG can access to oxygen in air. The VEG can be partially decomposed due to the lack of heat energy because the little heat from exothermic reaction after the disappearance of the flame. Finally, the container (c) with the curvature 0.17 cm^−1^ is suitable for the formation of precipitate and adequate oxygen and proper heat energy, which can ensure the sufficient pyrolysis of VEG to form VO_2_ (M).

**Figure 7:**
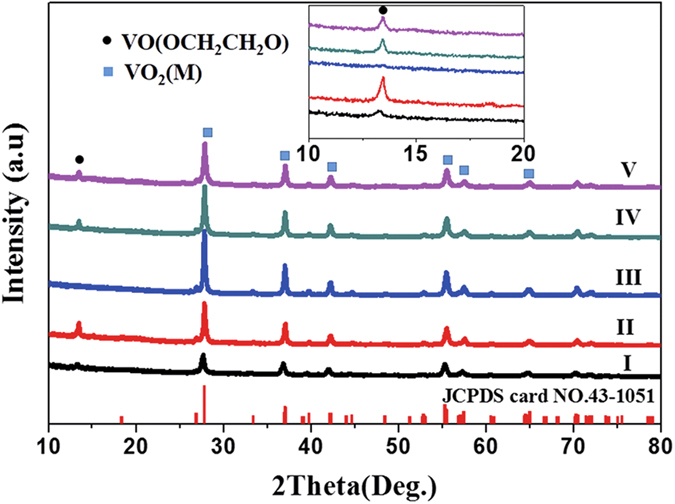
XRD patterns of prepared sample in container c with various volume of reaction solution: (I) 40 mL; (II) 60 mL; (III) 100 mL; (IV) 140 mL; (V) 200 mL.

Except for curvature, the volume of reaction solution is another factor to influence the pyrolysis. Thus, the experiments of the reaction solution with different volumes in the container (c) are carried out. The corresponding XRD patterns are shown in Fig. 7. It can be found that there is a diffraction peak of VEG at 2θ = 13.6° when the solution volume is at 40 mL and the appearance of diffraction peak of VEG could result from the lack of heat energy for VEG thin layer as the above mentioned. Whereas the solution volume is increased to 200 mL, a thick precipitate layer is observed. Similarly, the incomplete pyrolysis of VEG results in the appearance of diffraction peak of VEG, which is the same as that of the 100 ml reaction solution in container (a). As for diffraction peak at 2θ = 27.8° for VO_2_ (M), whether or not the volume of reaction solution is increasing or decreasing, the intensity become apparently weak compared with the solution of 100 mL in container (c). As a result, the crystallinity of VO_2_ (M) is optimal under the conditions of 100 mL solution in container (c). The VO_2_ powder obtained at the optimal condition is used for the subsequent tests and analyses.

### Analysis of TEM images for the obtained VO_2_

**Figure 8:**
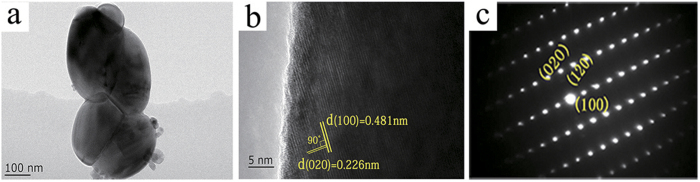
(**a**) TEM image of VO_2_ (M) nanoparticles; (**b**) HRTEM image and (**c**) SAED pattern of the an individual grain, respectively.

The size of the nanoparticles are measured by TEM images (Fig. 8a), which is close to the result shown in the SEM image (Fig. 3 (f)). Figure 8b shows the representative HRTEM image of individual nanoparticles. The interplanar distances of 0.483 nm and 0.226 nm matches well with the d_(100)_ and d_(020)_ spacings of monoclinic VO_2_ (M) structure, respectively. Furthermore, the measured lattice-plane angle is 90°, which is consistent with the result calculated from VO_2_ (M) crystallographic parameters, providing the microscopic evidence for the formation of VO_2_ (M)^13^. According to the SAED pattern (Fig. 8c), the independent and bright diffraction spots indicate that the VO_2_ (M) nanoparticles present good crystallinity. The dots are indexed to (100), (020) planes of VO_2_ (M), which are consistent with the result of HRTEM image.

### Analysis of the XPS spectrum for the obtained VO_2_

**Figure 9:**
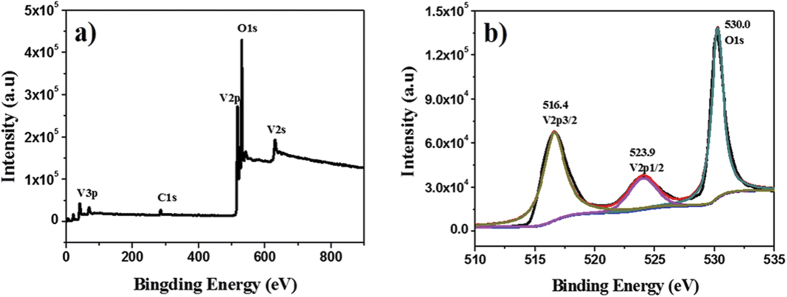
(**a**) XPS survey spectrum of the obtained VO_2_ (M) sample and (**b**) the High resolution XPS (HRXPS) for V2p and O1s region.

The XPS spectrum of the obtained products is presented in Fig. 9. The binding energies obtained in the XPS analysis were corrected for specimen charging by referencing the C1_s_ line to 284.6 eV. There are only three elements containing carbon, vanadium and oxygen in the XPS survey spectrum (Fig. 9a), where the carbon peak is attributed to surface contamination. Two peaks at 516.4 and 523.9 eV in the high-resolution XPS spectra (Fig. 9b) are associated with the spin-orbit splitting of V2_p3/2_ and V2_p1/2_, which are well in agreement with V^4+^ in the literature^25^. In addition, it has been established that the oxidation state of vanadium oxides can be determined by the difference of binding energy (Δ) between the O1s and V2_p3/2_ level. The Δ(O1_s_ -V2_p3/2_) value of as-obtained products is 13.6 eV that matches well with the result reported^26^. It indicates the vanadium of the sample is V^4+^ without other oxide state. Especially, the absence of V^5+^ may be attributed to the protection (such as H_2_ and CO_2_) generated in the combustion process^18^. Thus, XRD, TEM and XPS spectra confirm the high quality of the as-obtained VO_2_ (M) nanoparticles.

### The optical properties of the VO_2_ composite film

The integral visible transmittance and solar transmittance of VO_2_-based composite film were obtained based on the measured spectra using the following equation:


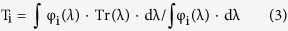


where Tr (λ) denotes the transmittance at wavelength λ, i denotes ‘vis’ or ‘sol’ for the calculations, φvis is the standard luminous efficiency function for the photopic vision, and φsol is the solar irradiance spectrum for the air mass 1.5 (corresponding to the sun standing 37° above the horizon)^27^. The modulation ability of 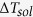 is defined as the difference of *T*_*sol*_ between 25 and 100 °C.

**Figure 10:**
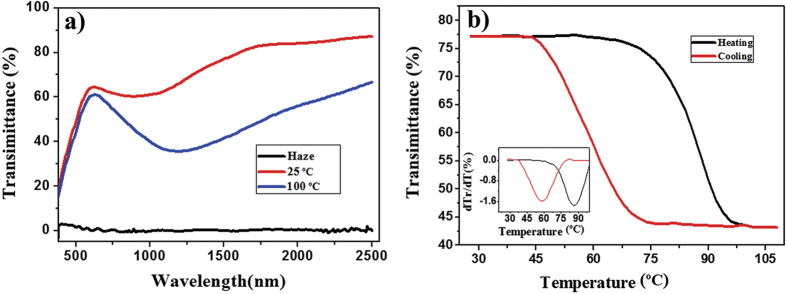
Thermochromic performance for the VO_2_-based composite film: (**a**) the transmission spectra from 380 to 2500 nm at 25 and 100 °C and the related haze; (**b**) the hysteresis loop of the composite film from 28 to 110 °C at the fixed wavelength of 1500 nm.

There are several crucial factors including 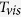, 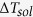, T_c_ and haze for VO_2_-based smart thin film. The much higher 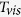, 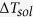, and lower T_c_, haze are hopefully obtained. In the past few years, it has been calculated that the VO_2_ nanoparticles are randomly and uniformly dispersed into transparent medium, of which the films possess numerous specific advantages over VO_2_-based continuous films^28^. In the work, the VO_2_ nanoparticles present high purity and good crystallinity. Consequently, by dispersing the VO_2_ nanoparticles into transparent polymer and spinning coating, the film of VO_2_-based is fabricated. The transmission spectra and related haze of the obtained film are presented in Fig. 10a. It can be found that the VO_2_-based film obtained shows excellent optical and thermochromic properties. The luminous transmittance 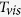 is up to 62.2% and the solar transmittance modulation 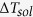 keeps 12.5%, which is consistent with the calculated result of the work^28^. Hence, the film shows better thermochromic properties compared with the previous strategies such as solution method^29^, physical vapour deposition (PVD)^30^. Moreover, the haze is 0.5% at 550 nm of the film which verifies significantly the uniformity of the thin film.

The hysteresis loop of the composite film at the fixed wavelength of 1500 nm is shown in Fig. 10b with embedding plot of dTr/dT-T. The temperature corresponding to the maximum of dTr/dT is defined as the phase transition temperature of the branch; T_1_ and T_2_ represent the phase transition temperature of heating and cooling branches, respectively. The phase transition temperature is defined as Tc = (T_1_+T_2_)/2^31^. In the past few years, it has been reported that the VO_2_ phase transition occurs through a nucleation and growth process affected by microstructure defects, particle sizes, grain boundaries^32^,^33^,^34^,^35^. From the Fig. 10b, it can be found that the phase transition temperature T_1_ = 58 °C and T_2_ = 86 °C. Hence, it can be calculated that T_c_ is equal to 72 °C, closing to 68 °C for the bulk of VO_2_ in the literature^36^. And a broad hysteresis loops widths in our paper is 28 °C, which is in line with the results that nanoparticles have fewer defects resulting in wide hysteresis loop in the literature reported by R. Lopez *et al*.^32^.

## Conclusion

In summary, monoclinic vanadium dioxide with pretty crystallinity is successfully fabricated by means of a facile combustion of precursor solution in the evaporating dish. The raw materials: NH_4_VO_3_, C_2_H_6_O_2_ and C_2_H_5_OH are inexpensive and the period of the preparation is relatively short in air without the protection of the extra inert gas. The curvature of container and the volume of solution are proved to be of significant to the fully pyrolysis of VEG and the formation of pure VO_2_ (M). Moreover, the composite foils (haze 0.5%) made from VO_2_ (M) particles displays excellent visible transmittance (up to 62.2%) and solar modulation ability (up to 12.5%). The present strategy exhibits the promising potential for smart window film with low cost and large-scale production.

## Methods

### Synthesis of VO_2_ (M)

All reagents were analytical grade and used without further purification. Firstly, a mixture of 2.0 g powders of NH_4_VO_3_ (99.0%, Aladdin reagent) and 75 mL of ethylene glycol (C_2_H_6_O_2_, Guangzhou Chemistry Reagent) in beaker was heated to 30–70 °C for 30 minutes with vigorous stirring. The transparent yellow solution was obtained after cooling to room temperature. Then the equal volume of ethanol was added to the above solution and the mixture was stirred for additional 30 minutes at the room temperature. Subsequently, the forming solution was placed in evaporating dish, and combusted directly in air. After completion, the black-blue powder on the inner wall of the evaporating dish was collected.

### Preparation of thermochronic films

To prepare VO_2_-based composite smart thermochronic film, VO_2_ (M) powder was mixed fully with the transparent polymer and stirred for several hours. The VO_2_ composite thin films were coated on the glass substrate by spinning with 2000 rpm.

### Characterization

X-ray powder diffraction (XRD) patterns were recorded on X’Pert Pro MPD diffractometer with Cu k_α_ radiation (λ = 0.154178 nm) using a current and voltage of 40 mA and 40 kV. Unless additional stated, all samples were measured at a scanning rate of 0.1°/2θs^−1^. For thermolysis analysis, the differential scanning calorimetry (DSC) experiments were performed using a NETZSCH thermal analyzer (DSC 204F1) under dry air flow in the range of 20–500 °C with a heating rate of 10 K min^−1^. A Hitachi S-4800 scanning electron microscope (SEM) at an acceleration voltage of 2 kV was used to acquire SEM and high-magnificent SEM images. X-ray photoelectron spectroscopy (XPS) measurements were performed on a Thermo Scientific ESCALAB 250 Xi X-ray phtotoelectron spectrometer. The morphology of the prepared particles was also observed by transmission electron microscopy (TEM, JEOL JEM-2010, Japan) operated at 200 kV. The attachment of selected area electron diffraction (SAED) of JEM-2010 was used to get the crystallographic information. To prepare the samples for TEM/HRTEM/SAED analysis, the nanostructures were dispersed in ethanol and then deposited onto 400 mesh carbon-coated Cu grids. The temperature evolution was detected by a thermocouple detector at various stages. Thermochromic properties of films were monitored on a Lambda 750 spectrophotometer equipped with a heating unit in the wavelength range of 380–2500 nm. Transmittance spectra before and after phase transition were recorded at 25 and 100 °C, respectively.

## Additional Information

**How to cite this article**: Cao, Z. *et al*. A simple and low-cost combustion method to prepare monoclinic VO_2_ with superior thermochromic properties. *Sci. Rep.* **6**, 39154; doi: 10.1038/srep39154 (2016).

**Publisher's note:** Springer Nature remains neutral with regard to jurisdictional claims in published maps and institutional affiliations.

## Figures and Tables

**Table 1:** The parameters of containers.

Container	a	b	c	d	e
Volume (mL)	100	150	250	500	500
Curvature radius (cm)	4.39	5.02	6.01	7.98	∞
Curvature (cm^−1^)	0.23	0.20	0.17	0.13	0

## References

[b1] Morin F. J. Oxides which show mental-to-insulator transition at the neel temperature. Phys. Rev. Lett. 3, 34–36 (1959).

[b2] Lee M. J. . Two series oxide resistors applicable to high speed and high density nonvolatile memory. Adv. Mater. 19, 3919–3923 (2007).

[b3] Liu M. . Terahertz-field-induced insulator-to-metal transition in vanadium dioxide metamaterial. Nature 487, 345–348 (2012). 22801506 10.1038/nature11231

[b4] Ye H. . The demonstration and simulation of the application performance of the vanadium dioxide single glazing. Sol. Energ. Mat. Sol. C. 117, 168–173 (2013).

[b5] Yang J. . Performance analyses of building energy on phase transition processes of VO_2_ windows with an improved model. Appl. Energ. 159, 502–508 (2015).

[b6] Zhao L. . A low cost preparation of VO_2_ thin films with improved thermochromic properties from a solution-based process. Thin Solid Films 543, 157–161 (2013).

[b7] Gaskell J. M. . Optimised atmospheric pressure CVD of monoclinic VO_2_ thin films with picosecond phase transition. Surf. Coat. Tech. 287, 160–165 (2016).

[b8] Livage J., Guzman G., Beteille F. & Davidson P. Optical properties of sol-gel derived vanadium oxide films. J. Sol-Gel. Sci. Techn. 8, 857–865 (1997).

[b9] Huang C., Chen l., Xu G. & Miao L. Sol–gel template synthesis and characterization of VO_2_ nanotube arrays. J Sol-Gel Sci. Techn. 63, 103–107 (2012).

[b10] Yuan N., Li J. & Lin C. Valence reduction process from Sol-Gel V_2_O_5_ to VO_2_ thin film. Appl. Surf. Sci. 191, 176–180 (2002).

[b11] Zheng C., Zhang J., Luo G., Ye J. & Wu M. Preparation of vanadium dioxide powders by thermolysis of a precursor at low temperature. J. Mater. Sci-Mater. El. 35, 3425–3429 (2000).

[b12] Peng Z., Jiang W. & Liu H. Synthesis and electrical properties of tungsten-doped vanadium dioxide nanopowders by thermolysis. J. Phys. Chem. C 111, 1119–1122 (2007).

[b13] Wu C. . Direct hydrothermal synthesis of monoclinic VO_2_ (M) single-domain nanorods on large scale displaying magnetocaloric effect. J. Mater. Chem. 21, 4509–4517 (2011).

[b14] Chen R. . One-step hydrothermal synthesis of V_1−x_W_x_O_2_ (M/R) nanorods with superior doping efficiency and thermochromic properties. J. Mater. Chem. A 3, 3726–3738 (2015).

[b15] Chen Z., Cao C., Chen S., Luo H. & Gao Y. Crystallised mesoporous TiO_2_ (A)–VO_2_ (M/R) nanocomposite films with self-cleaning and excellent thermochromic properties. J. Mater. Chem. A 2, 11874–11884 (2014).

[b16] Zhang L. . Phase and morphology evolution during the solvothermal synthesis of VO_2_ polymorphs. Inorg. Chem. Front. 3, 117–124 (2016).

[b17] Chen R. . Shape-controlled synthesis and influence of W doping and oxygen nonstoichiometry on the phase transition of VO_2_. Sci. Rep. 5, 14087, doi: 10.1038/srep14087 (2015). 26373612 PMC4650710

[b18] Wu C. . Direct confined-space combustion forming monoclinic vanadium dioxides. Angew. Chem. Int. Edit. 49, 134–137 (2010). 10.1002/anie.200905227 19943305

[b19] Li J. & Sun G. The synthesis bis (α-furylacrylic acid) oxovanadium (IV) complex. Chem. & Bioeng. 25, 10–11 (2008).

[b20] Nagabhushana G. P. & Chandrappa G. T. Facile solution combustion synthesis of monoclinic VO_2_: a unique and versatile approach. J. Mater. Chem. A 1, 11539–11542 (2013).

[b21] Zou J., Peng Y. & Lin H. A low-temperature synthesis of monoclinic VO_2_ in an atmosphere of air. J. Mater. Chem. A 1, 4250–4254 (2013).

[b22] Wang H. . Facile synthesis of hierarchical and porous V_2_O_5_ microspheres as cathode materials for lithium ion batteries. J. Colloid Interf. Sci. 418, 74–80 (2014). 10.1016/j.jcis.2013.12.011 24461820

[b23] Zhang H. . A cost-effective method to fabricate VO_2_ (M) nanoparticles and films with excellent thermochromic properties. J. Alloy. Compd. 636, 106–112 (2015).

[b24] Weeks C. . The one dimensional chain structures of vanadyl glycolate and vanadyl acetate. J. Mater. Chem. 13, 1420–1423 (2003).

[b25] Xu C., Ma L., Liu X., Qiu W. & Su Z. A novel reduction–hydrolysis method of preparing VO_2_ nanopowders. Mater. Res. Bull. 39, 881–886 (2004).

[b26] Silversmit G., Depla D., Poelman H., Marin G. B. & De Gryse R. Determination of the V2p XPS binding energies for different vanadium oxidation states (V^5+^ to V^0+^). J. Electron. Spectrosc. Relat. Phenom. 135, 167–175 (2004).

[b27] Chen Z. . VO_2_-based double-layered films for smart windows: Optical design, all-solution preparation and improved properties. Sol. Energ. Mat. Sol. C. 95, 2677–2684 (2011).

[b28] Li S. Y., Niklasson G. A. & Granqvist C. G. Nanothermochromics: Calculations for VO_2_ nanoparticles in dielectric hosts show much improved luminous transmittance and solar energy transmittance modulation. J. Appl. Phys. 108, 063525-1–063525-8 (2010).

[b29] Vernardou D., Pemble M. E. & Sheel D. W. The growth of thermochromic VO_2_ films on glass by atmospheric-pressure CVD: A comparative study of precursors, CVD methodology, and substrates. Chem. Vapor. Depos. 12, 263–274 (2006).

[b30] Kakiuchida H., Jin P. & Tazawa M. Control of thermochromic spectrum in vanadium dioxide by amorphous silicon suboxide layer. Sol. Energ. Mat. Sol. C. 92, 1279–1284 (2008).

[b31] Du J. . Significant changes in phase-transition hysteresis for Ti-doped VO_2_ films prepared by polymer-assisted deposition. Sol. Energ. Mat. Sol. C. 95, 469–475 (2011).

[b32] Lopez R., Haynes T. E., Boatner L. A., Feldman L. C. & Haglund R. F. Size effects in the structural phase transition of VO_2_ nanoparticles. Phys. Rev. B 65, 224113-1–224113-5 (2002).

[b33] Petit C., Frigerio J. M. & Goldmann M. Hysteresis of the metal–insulator transition of VO_2_: evidence of the influence of microscopic texturation. J. Phys-Condens. Mat. 11, 3259–3264 (1999).

[b34] Narayan J. & Bhosle V. M. Phase transition and critical issues in structure-property correlations of vanadium oxide. J. Appl. Phys. 100, 103524 (2006).

[b35] Whittaker L., Jaye C., Fu Z., Fischer D. A. & Banerjee S. Depressed Phase Transition in Solution-Grown VO_2_ Nanostructures. J. Am. Chem. Soc. 131, 8884–8894 (2009). 19505072 10.1021/ja902054w

[b36] Qazilbash M. M. . Mott transition in VO_2_ revealed by infrared spectroscopy and nano-imaging. Science 318, 1750–1753 (2007). 18079396 10.1126/science.1150124

